# Chronic Corticosterone Exposure Persistently Elevates the Expression of Memory-Related Genes in the Lateral Amygdala and Enhances the Consolidation of a Pavlovian Fear Memory

**DOI:** 10.1371/journal.pone.0091530

**Published:** 2014-03-11

**Authors:** Melissa S. Monsey, Lara M. Boyle, Melinda L. Zhang, Caroline P. Nguyen, Hope G. Kronman, Kristie T. Ota, Ronald S. Duman, Jane R. Taylor, Glenn E. Schafe

**Affiliations:** 1 Department of Psychology, Yale University, New Haven, Connecticut, United States of America; 2 Interdepartmental Neuroscience Program, Yale University, New Haven, Connecticut, United States of America; 3 Department of Psychiatry, Yale University, New Haven, Connecticut, United States of America; 4 Department of Psychology, The City University of New York, New York, New York, United States of America; 5 Center for Study of Gene Structure and Function, Hunter College, The City University of New York, New York, New York, United States of America; 6 The Graduate Center, The City University of New York, New York, New York, United States of America; Rosalind Franklin University, United States of America

## Abstract

Chronic exposure to stress has been widely implicated in the development of anxiety disorders, yet relatively little is known about the long-term effects of chronic stress on amygdala-dependent memory formation. Here, we examined the effects of a history of chronic exposure to the stress-associated adrenal steroid corticosterone (CORT) on the consolidation of a fear memory and the expression of memory-related immediate early genes (IEGs) in the lateral nucleus of the amygdala (LA). Rats received chronic exposure to CORT (50 μg/ml) in their drinking water for 2 weeks and were then titrated off the CORT for an additional 6 days followed by a 2 week ‘wash-out’ period consisting of access to plain water. Rats were then either sacrificed to examine the expression of memory-related IEG expression in the LA or given auditory Pavlovian fear conditioning. We show that chronic exposure to CORT leads to a persistent elevation in the expression of the IEGs Arc/Arg3.1 and Egr-1 in the LA. Further, we show that rats with a history of chronic CORT exposure exhibit enhanced consolidation of a fear memory; short-term memory (STM) is not affected, while long-term memory (LTM) is significantly enhanced. Treatment with the selective serotonin reuptake inhibitor (SSRI) fluoxetine following the chronic CORT exposure period was observed to effectively reverse both the persistent CORT-related increases in memory-related IEG expression in the LA and the CORT-related enhancement in fear memory consolidation. Our findings suggest that chronic exposure to CORT can regulate memory-related IEG expression and fear memory consolidation processes in the LA in a long-lasting manner and that treatment with fluoxetine can reverse these effects.

## Introduction

Stress has been strongly implicated in the development of numerous psychiatric disorders, including post-traumatic stress disorder (PTSD), an anxiety disorder characterized by intense fearful memory formation to cues associated with a traumatic event [1]-[4]. It is well established that the lateral nucleus of the amygdala (LA) is a critical region for the formation and storage of Pavlovian fear memories [5]. It is also known that the LA contains a rich supply of glucocorticoid receptors which bind glucocorticoids released in response to stressful stimuli [6], [7], rendering this brain region a prime target for the site of action of stress-induced modulation of fear memories [8].

Prolonged periods of stress or chronic exposure to glucocorticoids, including the adrenal steroid corticosterone (CORT), have been observed to promote multiple morphological and molecular alterations in brain regions associated with cognition and emotion. In rodent models, for example, prolonged exposure to stress or oral CORT has been shown to promote dendritic atrophy in the hippocampus, particularly in area CA3 [9]–[13], impair hippocampal neurogenesis [14]–[16], and significantly decrease the expression of several plasticity-related signaling proteins and genes in the hippocampus, including the activity of the extracellular signal-regulated kinase (ERK) and the cAMP-response element binding protein (CREB) and the expression of brain derived neurotrophic factor (BDNF) [17]–[21]. At a functional level, prolonged exposure to stress or oral CORT in rats has been shown to impair hippocampal synaptic plasticity [22], [23] and hippocampal-dependent memory formation [11], [23]–[26].

A remarkably different picture has emerged in the amygdala following prolonged exposure to chronic stress. In contrast to the hippocampus, exposure to chronic stress has been observed to promote dendritic hypertrophy in LA neurons [13], [27], increase dendritic spine density [28], [29] and to significantly increase the expression of BDNF [21]. These observations are consistent with the findings that a bout of chronic stress exposure can promote anxiety-like behavior in rats [27], [30] and enhance the formation of a Pavlovian fear memory when rats are trained and tested shortly after the stress exposure period [31], [32]. Further, one of the more notable, and perhaps clinically consequential, aspects of the effects of chronic stress on the amygdala is the persistence of its morphological and behavioral effects [8]. It is well established that the morphological and behavioral effects of chronic stress are reversible in the hippocampus following a period of recovery [21], [26], [27], [31]. In contrast, chronic stress has been shown to lead to persistent dendritic hypertrophy in LA neurons, a persistent increase in the expression of BDNF mRNA in the LA and persistent changes in anxiety-like behavior in rats that does not recover with time [21], [27].

Given the persistence of the effects of stress in the amygdala, it is surprising that few studies have systematically examined the long-term effects of chronic stress on amygdala-dependent memory formation. To that end, in the present study we examined the long-term effects of chronic exposure to oral CORT on the expression on plasticity-related signaling pathways and memory-related IEGs in the LA and on the consolidation of a Pavlovian fear memory. We show that chronic oral CORT exposure persistently elevates the expression of memory-related IEGs in the LA and enhances the consolidation of an auditory fear memory. Further, we show that each of these effects can be reversed following treatment with the selective serotonin-reuptake inhibitor (SSRI) fluoxetine. Collectively, our findings suggest that chronic stress, as modeled by a chronic oral CORT paradigm, leads to long-lasting alterations in amygdala-dependent learning and memory processes via alterations in memory-related genes in LA neurons, findings which may have relevance for understanding the neurobiological mechanisms underlying the development of psychiatric disorders such as PTSD that are characterized by unusually strong and persistent fear memories.

## Materials and Methods

### Subjects

Adult male Sprague-Dawley rats (Harlan), obtained at ∼3 months of age, were housed individually in plastic cages and maintained on a 12:12 hr light/dark cycle. Food and water were provided *ad libitum* throughout the experiments. All procedures were conducted under the guidelines provided in the National Institutes of Health *Guide for the Care and Use of Experimental Rats* and were approved by the Yale University Institutional Animal Care and Use Committee.

### Corticosterone exposure

A low-dose chronic oral CORT paradigm designed to mimic the effects of chronic mild stress was adapted from previous studies [17], [18], [20], [44]. Briefly, corticosterone (4-Pregnen-11β, 21-Diol-3, 20-Dione 21-Hemisuccinate; Steraloids, Inc) solution was made by dissolving it in tap water, bringing the pH to 12 and stirring for 1 hr. After CORT was in solution, the pH was brought down to 7.0–7.4 using HCl. The CORT solution was made fresh every 72 hours and bottles were weighed daily to measure rats' fluid consumption. For experiments examining the acute effects of chronic CORT exposure, rats received either water alone or CORT in their drinking water at a concentration of 50 μg/ml for 2 weeks followed by sacrifice. For experiments examining the long-lasting effects of chronic CORT exposure, rats received CORT in their drinking water at a concentration of 50 μg/ml for 2 weeks, followed by 6 days of CORT titration in which the concentration was reduced to 25 μg/ml for 3 days and then 12.5 μg/ml for an additional 3 days. Rats then received a ‘wash-out’ period during which they received access to regular water for 2 weeks. This low-dose chronic 14 d CORT exposure protocol has been shown to reliably increase serum CORT levels and to induce in rodent models a long-lasting phenotype of anhedonia, helplessness-like behavior and morphological changes in the hippocampus and prefrontal cortex similar to what has been observed with chronic behavioral stressors [17], [18], [20], [44], [62]. Importantly, it is the chronic nature of the model that appears to be critical; exposure to this low-dose oral CORT paradigm for 1 d has not been observed to promote molecular changes in the brain [19]. Further, unlike protocols that use higher doses of CORT [32], this protocol has been shown to be without significant effects on body weight during the CORT exposure period [18]. Levels of serum CORT have been observed to rapidly return to baseline following the CORT exposure period using this protocol [17], [18], and no long-term changes in either body or adrenal weights have been observed [18]. In our experiments, total fluid intake during the CORT exposure averaged ∼35–40 ml/day (∼1.75–2.0 mg CORT/day/rat), and was not observed to differ significantly between groups (water vs. CORT; *p* > 0.05; Figure S1a).

### Fluoxetine exposure

For the fluoxetine (FLX) treatment experiments, rats received exposure to 2 weeks of chronic oral CORT (50 μg/ml) followed by 6 days of CORT titration (25 μg/ml for 3 days; 12.5 μg/ml for 3 days) as described above. Following CORT titration, rats received a 3 week ‘wash-out’ period during which they were exposed to either regular water with 2% saccharin or fluoxetine in their drinking water (333 μl/ml). Fluoxetine hydrochloride (Matrix Scientific) was dissolved in tap water with 2% saccharin to mask the taste. Fluoxetine was made fresh weekly. Total fluid intake during the FLX exposure period averaged ∼30 ml/day (∼9.9 mg FLX/day/rat) and did not differ between groups (*p* > 0.05; Figure S1b).

### Western blotting experiments

To examine the expression of CORT-regulated proteins in the LA, rats were sacrificed either at the end of the 2 week exposure to 50 μg/ml CORT, the end of the 2 week water ‘wash-out’ period, or at the end of the 3 week period of treatment with fluoxetine. Rats were sedated using isoflurane and decapitated. Brains were frozen and stored at −80°C until processed. For Western blotting, punches were taken from the LA using a 1 mm punch tool (Fine Science Tools, Foster City, CA) from 400-μm-thick sections cut on a sliding freezing microtome. Punches were manually dounced in 100 μl of ice-cold hypotonic lysis buffer [10 mM Tris-HCl, pH 7.5, 1 mM EDTA, 2.5 mM sodium pyrophosphate, 1 mM phenylmethylsulfonyl fluoride, 1 mM β-glycerophosphate, 1% Igepal CA-630, 1% protease inhibitor cocktail (Sigma) and 1 mM sodium orthovanadate]. Sample buffer was immediately added to the homogenates, and the samples were boiled for 4 min. Homogenates were electrophoresed on 4–20% gels and blotted to Immobilon-P (Millipore, Bedford, MA). Western blots were blocked in 5% milk in TTBS buffer (50 mM Tris-HCl, pH 7.5, 150 mM NaCl, and 0.05% Tween 20) then incubated with anti-phospho ERK (1∶1K; Cell Signaling), anti-total ERK (1∶1K; Cell Signaling), anti-acetyl histone H3 (1∶3K; Millipore), anti-total histone H3 (1∶5K; Millipore), anti-Arc/Arg3.1 (1∶1K; Santa Cruz), anti-Egr-1 (1∶1K; Santa Cruz), anti-BDNF (1∶600; Millipore), anti-synaptophysin (1∶5K; Dako), or anti-GluR1 (1∶1K; Abcam) antibody. Blots were then incubated with anti-rabbit or anti-mouse conjugated to horseradish peroxidase (1∶20K; Cell Signaling) and developed using West Dura chemiluminescent substrate (Pierce). GAPDH (1∶5K; Abcam) was used as a loading control for all Western blotting experiments. Optical densities of the bands were analyzed using NIH Image software. For analysis of ERK and histone H3 proteins, optical densities for total ERK or total H3 were first normalized to GAPDH. Phospho-ERK or acetyl-H3 was then normalized to total ERK or total H3 for each sample and expressed as a percentage of that in the water alone control group. Optical densities for all other proteins were normalized to GAPDH and expressed as a percentage of the water alone control group.

### Elevated plus maze

Prior to fear conditioning, rats were tested in the elevated plus maze (EPM) to assess the effect of a history of chronic CORT exposure and the effects of subsequent FLX treatment on unlearned fear/anxiety-like responses. The EPM was performed essentially as previously described [63]. Briefly, each rat was placed at the center of the maze, and its behavior was recorded onto videotape for a period of 10 min. The total time each rat spent in the open arms and the numbers of entries into either the open or closed arms was recorded across the 10 min period by an observer blind to experimental conditions. The task was carried out in a small, dimly lit room (100LUX).

### Fear conditioning

Rats were habituated to handling and to the conditioning chambers for 2 days prior to training. On the training day, rats were fear conditioned with 2 tone-shock pairings consisting of a 20 sec, 5 kHz, 75 dB tone that co-terminated with a 1 sec, 0.25 mA foot shock (ITI = 120 sec). Testing for short-term memory (STM) and long-term memory (LTM) occurred at 2 and 24 hrs following training, respectively. For each test, rats were placed in a distinct environment that was dark and consisted of a flat black plastic floor that had been washed with a peppermint-scented soap. The STM test consisted of presentation of 3 conditioned stimulus (CS) tones to minimize extinction. The LTM test consisted of exposure to 10 CS tones. Freezing behavior, defined as a lack of all movement with the exception of that required for respiration, was recorded onto video tape during all testing sessions and scored by hand by an experimenter who was blind to the experimental conditions. Freezing to each 20 sec tone was expressed as a percentage of the total CS presentation time and data were analyzed with repeated-measures ANOVA. Differences were considered significant if *p* < 0.05.

## Results

### Chronic exposure to CORT enhances the expression of intracellular signaling pathways, memory-related IEGs and synaptically-localized proteins in the lateral amygdala

**Figure 1:**
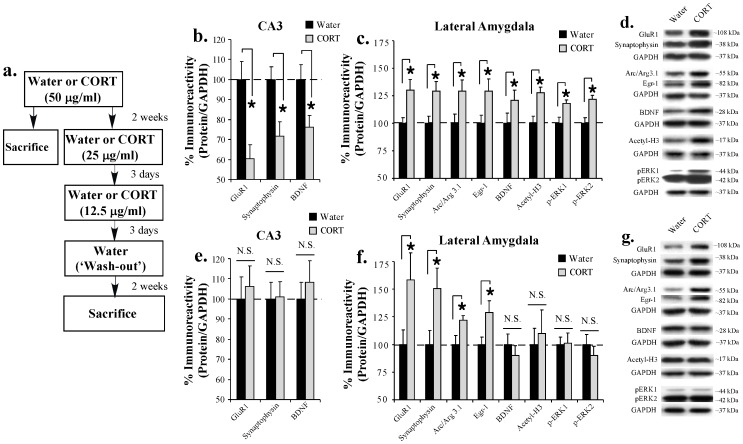
Chronic exposure to CORT persistently enhances the expression of memory-related IEGs and synaptically-localized proteins in the LA. (**A**) Schematic of the behavioral protocol. Rats received either Water or CORT in their drinking water (50 μg/ml) for 2 weeks. Half the rats were sacrificed at the end of CORT exposure period. The other half was titrated off the CORT (25 μg/ml, 12.5 μg/ml) and given a 2 week period of exposure to water alone (‘wash-out’) prior to being sacrificed. (**B**) Mean (±SEM) immunoreactivity for GluR1, synaptophysin and BDNF (Water: n = 9; CORT: n = 9) from area CA3 in rats sacrificed on the last day of CORT exposure. (**C**) Mean (±SEM) immunoreactivity for GluR1 (Water: n = 9; CORT: n = 9), synaptophysin (Water: n = 9; CORT: n = 9), Arc/Arg3.1 (Water: n = 9; CORT: n = 9), Egr-1 (Water: n = 9; CORT: n = 9), BDNF (Water: n = 9; CORT: n = 9), acetyl-H3 (Water: n = 7; CORT: n = 9), phospho-ERK 1 and phospho-ERK 2 (Water: n = 8; CORT: n = 9) from the LA in rats sacrificed on the last day of CORT exposure. (**D**) Representative Western blots for each protein in (C). (**E**) Mean (±SEM) immunoreactivity for GluR1, synaptophysin, and BDNF (Water: n = 9; CORT: n = 9) from area CA3 in rats sacrificed on the last day of the wash-out period. (**F**) Mean (±SEM) immunoreactivity for GluR1 (Water: n = 9; CORT: n = 8), synaptophysin (Water: n = 9; CORT: n = 9), Arc/Arg3.1 (Water: n = 9; CORT: n = 8), Egr-1 (Water: n = 9; CORT: n = 8), BDNF (Water: n = 9; CORT: n = 9), acetyl-H3 (Water: n = 9; CORT: n = 8), phospho-ERK 1 and phospho-ERK 2 (Water: n = 9; CORT: n = 8) from the LA in rats sacrificed on the last day of the wash-out period. (**G**) Representative Western blots for each protein in (F). For each experiment, protein levels have been normalized to GAPDH levels for each sample and expressed as a percentage of the Water group. (*) *p* < 0.05 relative to Water group.

As an initial step toward asking whether chronic exposure to CORT might promote alterations in the expression of plasticity-related proteins the LA that, in turn, regulate the consolidation of a fear memory, we first examined the expression of memory-related and synaptically-localized proteins in the LA following 2 weeks of chronic oral CORT exposure. Previous work from our lab and others has shown that the consolidation of a Pavlovian fear memory requires phosphorylation of the extracellular signal-regulated kinase (ERK), the acetylation of histone H3, and the expression of the immediate-early genes (IEGs) activity-regulated cytoskeletal-associated protein (Arc/Arg3.1), early growth response protein 1 (Egr-1) and BDNF in the LA [33]–[42]. In this first experiment, we examined the expression of each of these memory-related proteins in the LA on the final day of a 2-week chronic exposure to CORT (Figure 1a). We also examined the expression of the synaptically-localized proteins GluR1 and synaptophysin as an assay for whether chronic exposure to CORT may promote morphological (e.g. spine) changes in the LA that have previously been observed following exposure to a chronic behavioral stressor [13], [28], [29]. For a comparison with previous work that has documented stress- and CORT-related changes in neuronal morphology and BDNF expression in the hippocampus [9]–[13], [21], we also examined the expression of GluR1, synaptophysin and BDNF in samples from hippocampal area CA3 in the same animals.

Rats received either plain water or CORT (50 μg/ml) in their drinking water for 14 consecutive days and were sacrificed on the last day of CORT exposure (Figure 1a). Western blot analysis from punches taken from hippocampal area CA3 revealed that a 2 week exposure to CORT resulted in a significant downregulation of GluR1 [t_(16)_  =  3.47, *p* < 0.01], synaptophysin [t_(16)_  =  2.95, *p* < 0.01], and BDNF [t_(16)_  =  2.52, *p* < 0.05] proteins (Figure 1b). In contrast, and consistent with previous reports of increased dendritic length and branch points and spine number in LA neurons following a chronic behavioral stressor [13], [28], [29], we observed a significant upregulation in synaptophysin [t_(16)_  =  2.63, *p* < 0.05] and GluR1 [t_(16)_  =  2.66, *p* < 0.05] protein expression in the LA (Figure 1c). Further, Western blot analysis revealed that chronic CORT exposure resulted in a significant upregulation of phospho-ERK1 [t_(15)_  =  2.74, *p* < 0.05], phospho-ERK2 [t_(15)_  =  3.48, *p* < 0.01], acetylated histone H3 [t_(14)_  =  3.52, *p* < 0.05], Arc/Arg3.1 [t_(16)_  =  2.15, *p* < 0.05], Egr-1 [t_(16)_  =  2.23, *p* < 0.05] and BDNF [t_(16)_  =  2.27, *p* < 0.05] protein expression in the LA (Figure 1c). We observed no significant differences in the expression of total-ERK or total-H3 protein (ERK1: t_(15)_  =  0.40, *p* > 0.05; ERK2: t_(15)_  =  0.42, *p* > 0.05; H3: t_(14)_  =  0.78, *p* > 0.05) or in the loading control GAPDH between groups in any of our assays.

### Chronic exposure to CORT persistently enhances the expression of memory-related IEGs and synaptically-localized proteins in the lateral amygdala

In our next series of experiments, we examined the persistence of the CORT-related alterations in the expression of synaptically-localized and memory-related proteins in hippocampal area CA3 and the LA. Rats received chronic exposure to either plain water or CORT (50 μg/ml) in their drinking water for 14 days, followed by an additional 6 days of CORT titration (25 μg/ml for 3 days, 12.5 μg/ml for 3 days). Rats then received a ‘wash-out’ period consisting of exposure to regular water for an additional 2 weeks prior to sacrifice (Figure 1a).

Consistent with previous findings showing a reversal in stress-related morphological changes and in the expression of BDNF mRNA in area CA3 following a recovery period [21], [26], [27], [31], we observed no significant changes in either GluR1 [t_(16)_  =  0.42, *p* > 0.05], synaptophysin [t_(16)_  =  0.10, *p* > 0.05], or BDNF [t_(16)_  =  0.60, *p* > 0.05] proteins in area CA3 in rats with a history of chronic CORT exposure (Figure 1e). In contrast, and consistent with previous findings showing a persistent enhancement in dendritic length and branch points in the LA following exposure to a chronic behavioral stressor [27], we observed that rats with a history of chronic CORT exposure exhibited enhanced expression of both GluR1 [t_(15)_  =  2.05, *p*  =  0.05] and synaptophysin [t_(16)_  =  2.24, *p* < 0.05] in the LA (Figure 1f). Remarkably, we also observed a persistent increase in the expression of the IEGs Arc/Arg3.1 [t_(15)_  =  2.20, *p* < 0.05] and Egr-1 [t_(15)_  =  2.29, *p* < 0.05] that was not associated with persistently elevated levels of phospho-ERK1 [t_15)_  =  0.11, *p* > 0.05], phospho-ERK2 [t_(15)_  =  0.21, *p* > 0.05] or acetyl-H3 [t_(15)_  =  0.39, *p* > 0.05] protein expression in the LA (Figure 1f). In contrast to Arc/Arg3.1 and Egr-1, we observed no persistent increase in the expression of BDNF protein in the LA (t_(16)_  =  0.71, *p* > 0.05; Figure 1f). Levels of total ERK [ERK1: t_(15)_  =  0.10, *p* > 0.05; ERK2: t_(15)_  =  0.79, *p* > 0.05]; total-H3 [t_(15)_  =  0.05, *p* > 0.05] or the loading control GAPDH did not differ between groups in any of the experiments.

Collectively, our findings are consistent with those of previous reports of long-lasting dendritic hypertrophy in the LA following chronic behavioral stress [13], [27]. Further, given that these experiments were performed two weeks following the last exposure to CORT, our findings suggest that a history of chronic exposure to CORT can regulate memory-related IEG expression in the LA in a long-lasting manner.

### Rats with a history of chronic CORT exposure exhibit enhanced consolidation of a Pavlovian fear memory

In the preceding experiments, we showed that chronic exposure to CORT elevates the expression of the memory-related IEGs Arc/Arg3.1 and Egr-1 in the LA, and that the expression of these proteins remains elevated in a persistent manner long after the exposure to CORT has ended. Given that both Arc/Arg3.1 and Egr-1 have been implicated in fear memory consolidation processes in the LA [33], [36], in our next set of experiments we asked whether a history of chronic exposure to CORT can enhance the consolidation of a Pavlovian fear memory. Previous studies using both behavioral stressors and chronic exposure to oral CORT have observed enhancements in auditory and contextual fear memory shortly after the stress/CORT exposure period [31], [32], but see [43], findings that are consistent with our observations that a period of chronic CORT exposure can elevate the expression of plasticity-related signaling pathways in the LA. To date, however, few studies have examined whether a history of chronic stress or oral CORT exposure can modify the acquisition or consolidation of an amygdala-dependent fear memory.

**Figure 2:**
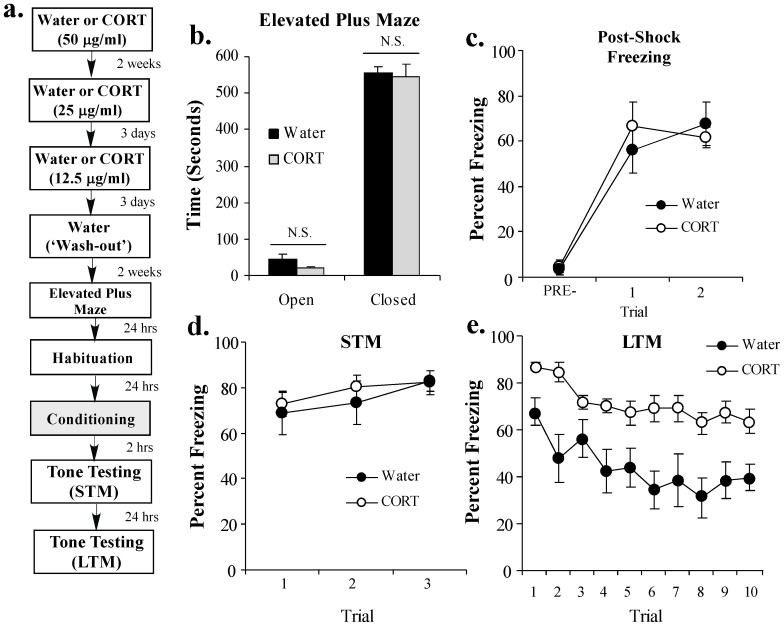
A history of chronic CORT exposure enhances the consolidation of a Pavlovian fear memory. (**A**) Schematic of the behavioral protocol. Rats received either Water (n = 7) or CORT (n = 9; 50 μg/ml) for 2 weeks followed by CORT titration (25 μg/ml, 12.5 μg/ml) and a 2 week water ‘wash-out’ period. At the end of the wash-out period, rats were tested in the elevated plus maze (EPM). Rats were then fear conditioned with 2 tone-shock pairings and tested for STM (2 hr later) and LTM (24 hr later). (**B**) Mean (±SEM) time spent exploring the open and closed arms of the EPM in each group. (**C**) Mean (±SEM) post-shock freezing scores in each group following each conditioning trial. (**D**) Mean (±SEM) STM scores across each trial in each group. (**E**) Mean (±SEM) LTM scores across each trial in each group.

In our experiments, rats received exposure to either plain water or CORT (50 μg/ml) in their drinking water for 2 weeks, followed by an additional 6 days of CORT titration (25 μg/ml for 3 days, 12.5 μg/ml for 3 days) as before. Rats then received a ‘wash-out’ period for an additional 2 weeks consisting of exposure to regular water. Following the wash-out period, all rats were tested in the elevated plus maze (EPM) to assay the effects of a history of chronic CORT exposure on unlearned fear/anxiety-like behavior. Following the EPM test, rats were habituated to the fear conditioning chamber for 2 days followed by fear conditioning consisting of 2 tone-shock pairings. Short-term and long-term memory tests were performed 2 and 24 hrs later, respectively, in a distinct context (Figure 2a).

Consistent with previous reports [18], [44], we observed no significant effects of prior chronic exposure to CORT in the EPM (Figure 2b). No statistically significant differences were observed in the total time spent in either the open [t_(14)_  =  1.56, *p* > 0.05] or closed [t_(14)_  =  0.26, *p* > 0.05] arms of the maze (Figure 2b). Further, we observed no significant differences in the total number of entries into the open [t_(14)_  =  1.45, *p* > 0.05] or closed [t_(14)_  =  1.46, *p* > 0.05] arms of the maze (data not shown).

In our fear conditioning experiments, we observed no differences in post-shock freezing (PSF) during training (Figure 2c). The ANOVA for PSF scores revealed nonsignificant effects for group [F_(1,14)_  =  0.07, *p* > 0.05] and the group by trial interaction [F_(2,28)_  =  0.70, *p* > 0.05], but a significant effect for trial [F_(2,28)_  =  45.03, *p* < 0.001]. Further, water and CORT-treated rats exhibited comparable levels of freezing during the STM test (Figure 2d). The ANOVA for STM scores revealed nonsignificant effects for group [F_(1,14)_  =  0.27, *p* > 0.05], trial [F_(2,28)_  =  2.04, *p* > 0.05], and the group by trial interaction [F_(2,28)_  =  0.20, *p* > 0.05]. Therefore, a history of chronic CORT exposure does not interfere with shock sensitivity (as assayed by PSF) or the acquisition or short-term storage of an auditory fear memory. During the LTM test, however, rats with a history of chronic CORT exposure exhibited a significantly higher level of fear memory retention relative to water controls (Figure 2e). The ANOVA for LTM scores revealed significant effects for group [F_(1,14)_  =  31.65 *p* < 0.001] and trial [F_(9,126)_  =  5.66, *p* < 0.001], and a nonsignificant group by trial interaction [F_(9,126)_  =  0.65, *p* > 0.05].

Collectively, our findings suggest that a prior history of chronic oral CORT exposure is able to enhance not only the expression of memory-related IEGs in the LA in a persistent manner, but also the consolidation of an auditory Pavlovian fear memory; that is, LTM is enhanced, while STM is unaffected.

### Fluoxetine treatment following chronic CORT exposure reverses the persistent enhancement in memory-related IEG expression in the LA

**Figure 3:**
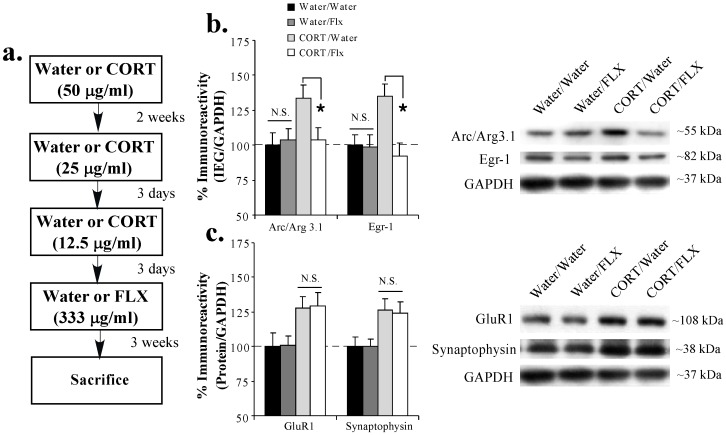
Fluoxetine treatment following chronic CORT exposure reverses the persistent enhancement in memory-related IEG expression in the LA. (**A**) Schematic of the behavioral protocol. Rats received either Water or CORT (50 μg/ml) for 2 weeks followed by CORT titration (25 μg/ml, 12.5 μg/ml). Rats then received 3 weeks of either Water or fluoxetine (FLX; 333 μg/ml) and were then sacrificed. (**B**) Mean (±SEM) immunoreactivity for Arc/Arg3.1 (Water/Water: n = 8; Water/Flx: n = 8; CORT/Water: n = 8; CORT/Flx: n = 7) and Egr-1 (Water/Water: n = 8; Water/Flx: n = 8; CORT/Water: n = 8; CORT/Flx: n = 8) in the LA for each group. (**C**) Mean (±SEM) immunoreactivity for GluR1 and synaptophysin (Water/Water: n = 8; Water/Flx: n = 8; CORT/Water: n = 8; CORT/Flx: n = 8) in the LA for each group. Representative Western blots for all proteins are shown in the inset. For each experiment, protein levels have been normalized to GAPDH levels for each sample and expressed as a percentage of the Water/Water group. (*) *p* < 0.05 relative to Water/Water group.

Previous work has shown that treatment with the SSRI fluoxetine following a period of chronic oral CORT exposure can normalize CORT-related molecular changes in the hippocampus [18]. In our next series of experiments, we asked whether treatment with fluoxetine can reverse the effects of a history of chronic CORT on the persistent expression of memory-related IEGs and synaptically-localized proteins in the LA. Rats received either plain water or chronic exposure to CORT (50 μg/ml) in their drinking water for 2 weeks followed by 6 days of CORT titration (25 μg/ml for 3 days, 12.5 μg/ml for 3 days) as before. Following CORT titration, each group was divided into two additional groups that received either plain water or fluoxetine (FLX; 333 μg/ml) in their drinking water for an additional 3 weeks resulting in the following 4 groups: Water/Water, Water/FLX, CORT/Water, CORT/FLX. On the last day of water or fluoxetine treatment, rats were sacrificed and brains were processed for protein analysis (Figure 3a).

Consistent with our previous findings, Western blot analysis revealed that a prior chronic exposure to CORT led to significant elevations in the expression of Arc/Arg3.1, Egr-1, GluR1 and synaptophysin in the LA (Water/Water vs. CORT/water groups; Figure 3b). Remarkably, a 3 week treatment with FLX was observed to reverse the effects of CORT on memory-related IEG expression in the LA (CORT/Water vs. CORT/FLX groups; Figure 3b). The ANOVAs for the IEGs revealed a significant effect for group [Arc/Arg3.1: F_(3,27)_  =  3.11, *p* < 0.05; Egr-1: F_(3,28)_  =  4.97, *p* < 0.01], with the CORT/Water groups differing significantly from Water/Water, Water/FLX, and CORT/FLX groups (*p* < 0.05; Duncan's test). Treatment with FLX alone (Water/Water vs. Water/FLX) was observed to have no effect on IEG expression in the LA (*p* > 0.05).

Interestingly, treatment with FLX was observed to have no effect on the persistent enhancement of either GluR1 or synaptophysin the LA (Figure 3c). The ANOVAs for the synaptically-localized proteins revealed a significant effect for group [GluR1: F_(3,28)_  =  3.61, *p* < 0.05; synaptophysin: F_(3,28)_  =  3.88, *p* < 0.05]. Duncan's post-hoc t-tests revealed significant differences between the CORT/Water and CORT/FLX groups relative to the Water/Water and Water/FLX groups (*p* < 0.05), while treatment with FLX alone (Water/Water vs. Water/FLX) was found to have no effect (*p* > 0.05). No significant difference between the CORT/Water and CORT/FLX groups was observed (*p* > 0.05).

Collectively, our findings indicate that chronic treatment with the SSRI fluoxetine following a period of chronic CORT exposure can effectively reverse the persistent CORT-induced changes in memory-related IEG expression in the LA.

### Fluoxetine treatment following chronic CORT exposure reverses the CORT-induced enhancement of fear memory consolidation

**Figure 4:**
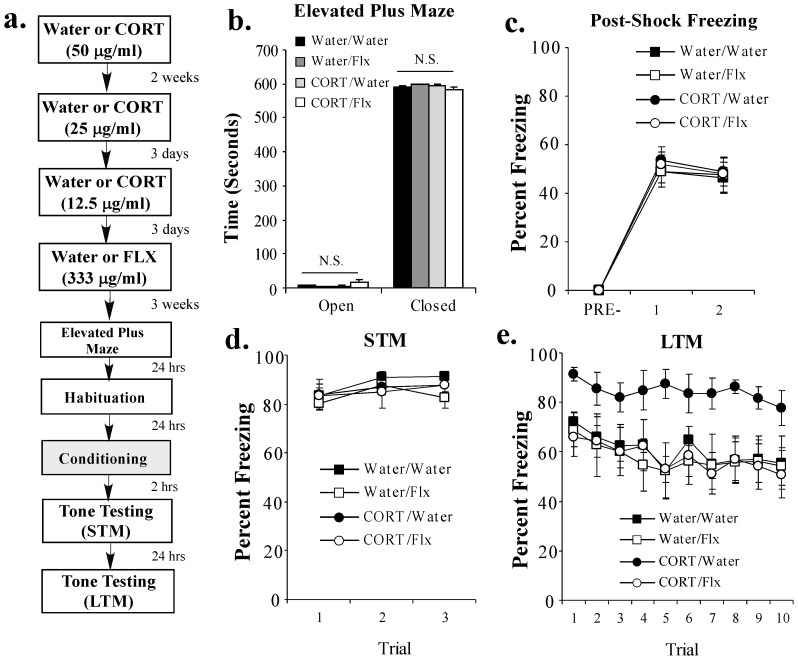
Fluoxetine treatment following chronic CORT exposure reverses the CORT-induced enhancement of fear memory consolidation. (**A**) Schematic of the behavioral protocol. Rats received either Water (n = 7) or CORT (n = 7; 50 μg/ml) for 2 weeks followed by CORT titration (25 μg/ml, 12.5 μg/ml). Rats then received 3 weeks of either Water (n = 6) or fluoxetine (FLX; n = 7; 333 μg/ml). At the end of the fluoxetine exposure period, rats were tested in the elevated plus maze (EPM). Rats were then fear conditioned with 2 tone-shock pairings and tested for STM (2 hr later) and LTM (24 hr later). (**B**) Mean (±SEM) time spent exploring the open and closed arms of the EPM in each group. (**C**) Mean (±SEM) post-shock freezing scores in each group following each conditioning trial. (**D**) Mean (±SEM) STM scores across each trial in each group. (**E**) Mean (±SEM) LTM scores across each trial in each group.

In our final experiment, we asked whether fluoxetine treatment following a history of chronic oral CORT exposure is capable of reversing the CORT-related enhancement in fear memory consolidation. Rats received either plain water or chronic exposure to CORT (50 μg/ml) in their drinking water for 2 weeks followed by CORT titration (25 μg/ml for 3 days, 12.5 μg/ml for 3 days) and an additional 3 weeks of treatment with either water or fluoxetine (333 μg/ml) as above. Following the FLX treatment period, rats in each of the 4 groups were tested in the EPM to assay the effects of CORT and FLX on unlearned fear/anxiety-like responses. Rats were then habituated to the conditioning chamber for 2 days prior to fear conditioning consisting of 2 tone-shock pairings and tested for both STM (at 2 hr) and LTM (at 24 hr) in a distinct context as before (Figure 4a). All rats remained on their respective treatment (water or FLX) throughout the duration of the behavioral experiments.

Similar to our findings above, we observed no significant effects of a prior chronic exposure to CORT in the EPM. We also observed that treatment with this dose of FLX alone had no significant effect on rats' performance in the EPM (Figure 4b). No statistically significant differences between groups were observed in the total time spent in either the open [F_(3,22)_  =  2.02, *p* > 0.05] or closed [F_(3,22)_  =  2.06, *p* > 0.05] arms of the maze (Figure 4b). Further, we observed no significant differences in the total number of entries into the open [F_(3,22)_  =  2.15, *p* > 0.05] or closed [F_(3,22)_  =  1.49, *p* > 0.05] arms of the maze (data not shown).

In our fear conditioning experiments, we observed no differences in post-shock freezing during training among the 4 groups (Figure 4c). The ANOVA for PSF scores revealed a significant effect for trial [F_(2,46)_  =  214.09, *p* < 0.001], but nonsignificant effects for group [F_(3,23)_  =  0.09, *p* > 0.05] and the group by trial interaction [F_(6,46)_  =  0.08, *p* > 0.05]. Further, each of the 4 groups exhibited comparable levels of freezing during the STM test (Figure 4d). The ANOVA for STM scores revealed nonsignificant effects for group [F_(3,23)_  =  0.54, *p* > 0.05], trial [F_(2,46)_  =  2.86, *p* > 0.05], and the group by trial interaction [F_(6,46)_  =  0.40, *p* > 0.05]. During the LTM test, however, the CORT/Water group demonstrated significantly higher levels of fear memory retention relative to all other groups (*p* < 0.05; Duncan's test; Figure 4e). The ANOVA for LTM scores revealed significant effects for group [F_(3,23)_  =  7.63, *p* < 0.01] and trial [F_(9,207)_  =  2.50, *p* < 0.001], but a nonsignificant group by trial interaction [F_(27,207)_  =  0.21, *p* > 0.05]. Importantly, there was no effect of this dose of FLX alone on the expression of fear memory in the Water/FLX group (*p* > 0.05).

Collectively, these findings indicate that treatment with the SSRI fluoxetine following a period of chronic oral CORT exposure can reverse not only the persistent elevation in memory-related IEG expression in the LA, but also the CORT-related enhancement in fear memory consolidation.

## Discussion

The effects of chronic stress and oral CORT administration have been widely studied at the behavioral, morphological and molecular levels, particularly in the hippocampus [9], [10], [12], [13], [17]–[20], [23]–[26], [31]. More recent work has focused on examining the effects of chronic behavioral stress in the amygdala, where long-lasting alterations in anxiety-like behavior and in the morphology of LA neurons have been observed [13], [21], [27]–[30]. While exposure to chronic stress or oral CORT have each been shown to enhance amygdala-dependent fear memory shortly after the stress/CORT period [31], [32], few studies have to date examined the long-lasting effects of a history of chronic stress or oral CORT on plasticity-related signaling pathways in the LA and amygdala-dependent memory processes. In the present study, we examined the long-lasting molecular and behavioral consequences of prior chronic oral CORT exposure, a pharmacological model of chronic stress with construct, face and predictive validity [17]–[20]. We found that a history of chronic oral CORT exposure leads to a persistent elevation in the expression of memory-related IEGs in the LA and to enhanced consolidation of an amygdala-dependent Pavlovian fear memory. Further, we show that each of these effects can be reversed by treatment with the SSRI fluoxetine.

In the hippocampus, the effects of chronic stress and oral CORT exposure have consistently been observed to promote dendritic atrophy [9]–[13] and a downregulation of memory-related signaling pathways and genes, including that of phosphorylated ERK, phosphorylated CREB and BDNF [17]–[21]. Consistent with these findings, we show that a 2-week exposure to oral CORT produces a downregulation in the expression of BDNF and the synaptically-localized proteins GluR1 and synaptophysin in hippocampal area CA3. Interestingly, while stress-induced morphological changes in the hippocampus have largely been observed to be transient [27], [31], the molecular changes have been observed to persist after a recovery period in some [17]–[20], but not all [21], studies. In our own experiments, we observed that the CORT-related downregulation in the expression of GluR1, synaptophysin, and BDNF in hippocampal area CA3 was transient. In the amygdala, we observed that chronic exposure to oral CORT promotes an upregulation of phosphorylated ERK, acetylated histone H3, and the expression of the IEGs Arc/Arg3.1, Egr-1 and BDNF in LA neurons. Consistent with previous work that has observed long-lasting dendritic hypertrophy in the LA following chronic behavioral stress [13], [27]–[29], these molecular changes were paralleled by an increase in the expression of the synaptically-localized proteins GluR1 and synaptophysin in the LA that persisted following the recovery period. Remarkably, while the CORT-induced enhancement in the expression of phospho-ERK and acetyl-H3 was observed to return to baseline levels following recovery from CORT exposure, the enhanced expression of the memory-related IEGs Arc/Arg3.1 and Egr-1 was observed to persist.

At the behavioral level, we observed that the consolidation of auditory Pavlovian fear conditioning was also enhanced by prior chronic oral CORT exposure; LTM was enhanced, whereas post-shock freezing and STM were unaffected. The finding that post-shock freezing and STM were unaffected makes it unlikely that our behavioral effects were due to altered tone or shock processing at the time of fear acquisition. Further, consistent with previous observations [18], [44], we observed no significant effect of prior chronic CORT exposure on unlearned fear/anxiety-like behavior. Collectively, our findings suggest that chronic oral CORT exposure can regulate the expression of memory-related IEGs in the LA in a long-lasting manner and promote enhanced consolidation of a Pavlovian fear memory acquired long after the CORT exposure period has ended. Notably, a related study using the same oral CORT exposure protocol found no significant effects on fear memory consolidation using a contextual fear conditioning paradigm [44]. However, contextual fear conditioning depends on both the amygdala and the hippocampus [45]–[47]. Thus, it is likely that enhanced and impaired consolidation processes at the level of the amygdala and hippocampus, respectively, driven by potentially opposing molecular alterations, may mask CORT-induced alterations in contextual fear memory consolidation [44]. In the present study, the use of an auditory fear conditioning paradigm may have allowed us to selectively observe a CORT-induced enhancement of amygdala-dependent fear memory consolidation.

What is the mechanism by which oral CORT persistently upregulates the expression of memory-related IEGs in the LA? One attractive hypothesis is that chronic CORT exposure promotes epigenetic alterations on memory-related genes in LA neurons, which result in long-lasting changes in the manner in which these genes are expressed. Epigenetic modifications, including alterations in chromatin structure and DNA methylation, have been widely implicated in memory and cognition and in brain regions associated with drug addiction and chronic stress, where they produce persistent alterations in gene expression that promote long-lasting behavioral phenotypes [48]–[51]. Chronic social defeat stress, for example, has been shown to promote significant increases in histone H3-K27 dimethylation, a repressive histone modification, on the promoters of BDNF transcripts III and IV [52]. In the nucleus accumbens (NAcc), chronic social defeat has been shown to significantly decrease the expression of histone deacetylase (HDAC) subtype 5, and mice that lack HDAC5 have been observed to exhibit enhanced depressive and anxiety-like behaviors [53]. Further, exposure to chronic social stress has been observed to lead to an increase in DNA methyltransferase (DNMT) subtype 3A mRNA expression in the NAcc, and virally-mediated overexpression DNMT3A in the NAcc has been shown to promote pro-depressive and anxiety-like behaviors [54]. These observations suggest that chronic stress can modify the chromatin structure and methylation patterns of genes in a regionally specific and persistent manner, which may in turn promote long-lasting behavioral phenotypes. Future studies will be required to examine epigenetic modifications in memory-related genes in the LA, including Arc/Arg3.1 and Egr-1, following chronic oral CORT exposure.

In our experiments, we observed that chronic treatment with the SSRI fluoxetine reversed both the persistent elevation in memory-related IEG expression in the LA and the enhanced consolidation of a fear memory in CORT-exposed rats. Fluoxetine is one of the most widely prescribed SSRIs for the treatment of a range of anxiety disorders, including PTSD [55]-[57], and accumulating evidence from animal models has suggested that treatment with SSRIs may be a useful therapeutic tool for the treatment of persistent and reoccurring traumatic memories [58]–[60]. In our experiments, we observed no effect of FLX alone (at this dose and route of administration) on memory-related gene expression in the LA and on fear memory acquisition or consolidation; FLX was only observed to reverse the enhancement of memory-related IEG expression in the LA and fear memory consolidation in rats exposed to chronic CORT. Notably, we observed no effect of FLX on the persistent CORT-induced increases in the synaptically-localized proteins GluR1 and synaptophysin in the LA. This finding is relevant in light of work suggesting that treatment with the atypical antidepressant (ADT) tianeptine during a period of chronic stress can prevent the stress-induced morphological changes in both hippocampal area CA3 and the LA [31], [61], while having no effect on the stress-induced enhancement of fear conditioning [31]. Together with our findings, these observations suggest that persistent morphological changes in the LA that are induced by chronic stress or oral CORT may not be casually related to enhanced memory formation and/or consolidation.

The mechanism by which FLX reverses CORT-related enhancements in fear memory consolidation and memory-related gene expression in the LA remains unknown, but may be related to the targeting of epigenetic processes in the LA. For example, chronic treatment with the ADT imipramine significantly decreases the expression of HDAC5 mRNA in the hippocampus and promotes a hyperacetylation of histone H3 on the promoter region of BDNF transcripts III and IV [52], providing a potential pro-transcriptional mechanism by which to counter the persistent dimethylation of histone H3-K27 on these BDNF transcripts. In contrast, chronic treatment with imipramine has been shown to lead to increased expression of HDAC5 in the NAcc [53]. These observations suggest that ADTs can promote epigenetic alterations on plasticity-related genes that compensate for the stress-related alterations in chromatin structure and DNA methylation. Future experiments will be necessary to examine the role of epigenetic mechanisms in the effects of FLX in the LA.

In summary, the findings of the present study suggest that a history of chronic stress, as modeled by a chronic oral exposure to CORT, can enhance fear memory consolidation processes and regulate memory-related IEG gene expression in the LA in a long-lasting manner, and that treatment with the SSRI fluoxetine can reverse these effects. The chronic oral CORT paradigm represents a useful animal model for investigation of amygdalar-regulated stress mechanisms that should help us to better understand the development of anxiety disorders such as PTSD that are characterized by unusually strong and persistent fear memories.

## Acknowledgments

We thank Dr. Shannon Gourley for helpful discussions about this work and assistance with the chronic corticosterone administration protocol.

## Supporting Information

Figure S1 **Fluid consumption during the CORT and FLX exposure periods.** (**A**) Fluid consumption (mL) was measured daily for all rats. Averages were taken for each group for each day of the chronic CORT exposure period (Water/Water: n = 7; Water/FLX: n = 6; CORT/Water: n = 7; CORT/FLX: n = 7; Days 1–14) and during the wash-out period (Days 15–20). (**B**) Average fluid consumption (mL) for each group for each day of the fluoxetine exposure period (Water/Water: n = 7; Water/FLX: n = 6; CORT/Water: n = 7; CORT/FLX: n = 7).
(TIF)
